# Photonic generation of ASK microwave signals with SSB format

**DOI:** 10.1007/s12200-023-00075-2

**Published:** 2023-07-24

**Authors:** Weilei Gou, Yuan Yu, Xinliang Zhang

**Affiliations:** 1 Wuhan National Laboratory for Optoelectronics and School of Optical and Electronic Information, Huazhong University of Science and Technology, Wuhan, 430074 China; 2 Optics Valley Laboratory, Wuhan, 430074 China

**Keywords:** Single sideband modulation, Amplitude shift keying, Microwave signal generation, Microwave communication

## Abstract

Optical beating is the usual approach to generation of microwave signals. However, the highest frequency achievable for microwave signals is limited by the bandwidths of optoelectronic devices. To maximize the microwave frequency with a limited bandwidth of a photodetector (PD) and relieve the bandwidth bottleneck, we propose to generate microwave signals with the single sideband (SSB) format by beating a continuous wave (CW) light with an optical SSB signal. By simply adjusting the frequency difference between the CW light and the carrier of the optical SSB signal, the frequency of the generated microwave SSB signal is changed correspondingly. In the experiment, amplitude shift keying (ASK) microwave signals with the SSB format are successfully generated with different carrier frequencies and coding bit rates, and the recovered coding information agrees well with the original pseudo random binary sequence (PRBS) of 2^7^ − 1 bits. The proposed approach can significantly relieve the bandwidth restriction set by optoelectronic devices in high-speed microwave communication systems.

**Graphical Abstract:**

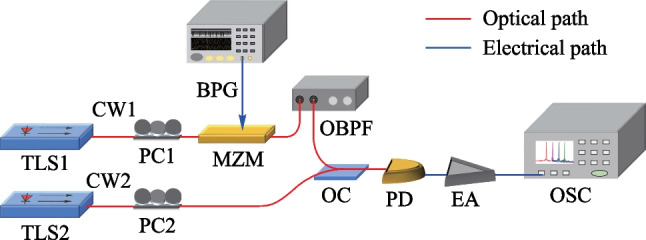

## Introduction

Digital modulation of microwave signals can be used on many scenarios, such as electronic warfare, wireless communication and modern radar systems [1–3]. As a basic digital modulation signal, amplitude shift keying (ASK) microwave signal is always used as the reference signal in communication systems [4]. Initially, ASK microwave signals were generated based on purely electronic techniques, such as radio frequency analog mixing and direct digital synthesis. However, the carrier frequency and coding bit rate of ASK microwave signals generated by electronic methods cannot meet the requirements of modern communication systems because of the bandwidth limitations of electronic systems. Photonic generation of ASK microwave signals has been considered as a promising approach due to its intrinsic advantages over electronical methods, such as high frequency, large bandwidth, and immunity to electromagnetic interference [5].

Frequency up‐conversion is a common method for generation of ASK microwave signals [6]. However, a strong frequency component is involved in the generated ASK microwave signal because of the beating between two optical carriers, which can cause interference at the detection stage. By using electro‐optical modulation [7] and optical pulse shaping [8], the strong frequency component can be suppressed. Nevertheless, it is still necessary to filter out the unwanted low-frequency or baseband components to reduce interference with other frequency bands. To solve these problems, phase modulation to intensity modulation conversion technique is proposed [9, 10].

Based on the above‐mentioned methods, ASK microwave signals are generated with double sideband (DSB) [11], optical carrier suppression (OCS) [12], and double sideband carrier suppression (DCS‐CS) modulations [13]. However, when an optical carrier is modulated to generate a DSB-modulated signal, the dispersion in single-mode fiber can cause severe power fading of the received microwave signals [14]. Besides, the highest microwave frequency after beating is significantly limited by the bandwidths of the optical DSB signal and optoelectronic devices, which significantly limits the microwave frequency bands that can be used. At the same time, the waveform of the signal with DSB format can be degraded because of the uneven amplitude and phase responses of optoelectronic devices [15]. Therefore, several schemes have been proposed to solve these problems using single sideband (SSB) modulation. These schemes include: using an optical filter to filter out one undesired sideband [16], using the stimulated Brillouin scattering (SBS) effect in the fiber to amplify one sideband [17], using vestigial sideband filtering combined with OCS to generate optical SSB signals [18], using an injection-locked semiconductor laser to amplify one modulation sideband [19], and combining a 90° hybrid coupler with a quadrature-biased dual-drive Mach–Zehnder modulator (DDMZM) [20]. These approaches are usually used to obtain optical SSB signals in optical communication systems, which can improve spectral efficiency, avoid signal degradation caused by the dispersion-induced power fading [20], and reduce the bit error rate. In these methods, using an optical filter to obtain optical SSB signal is a simple and effective approach because of the advantages of large bandwidth and stable frequency response. Besides optical communication systems, the SSB modulation is also desirable in microwave communication systems to promote spectral efficiency and signal fidelity.

In this paper, we propose to achieve a microwave SSB signal to maximize the microwave carrier frequency and spectral efficiency. By beating an optical SSB signal with another optical carrier, a microwave SSB signal is generated. In the experiment, ASK microwave SSB signals with different carrier frequencies and bit rates are successfully generated. As two examples, the carrier frequency and coding rate are switched from 30 to 20 GHz and from 10 to 5 Gb/s, respectively. Compared with the microwave DSB signal generation, a higher microwave frequency can be obtained using the proposed approach, thus the generated microwave signal can be transmitted via higher frequency bands. On the other hand, this approach can relieve the bandwidth requirement of optoelectronic devices in microwave communication systems. Optoelectronic devices with a smaller bandwidth can be used and the cost is consequently reduced. This advantage is more evident when the bandwidth of the microwave signal is large.

## Principle

**Fig. 1:**
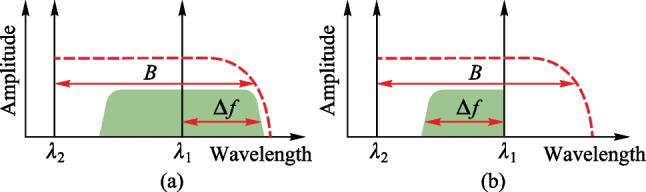
Optical spectra of one optical carrier and one intensity modulated signals with **a** DSB and **b** SSB format. The spectrum of the intensity modulated optical signal (green zone) and the required minimum bandwidth of optoelectronic devices (red dashed curve) are also marked

Figure 1a and b show the optical spectra of one optical carrier and one intensity modulated signal with DSB and SSB format, respectively. The spectrum of the intensity modulated optical signal is shown in the green zone in Fig. 1, where *λ*_1_ and *λ*_2_ are the wavelengths of two optical carriers and the corresponding frequencies are *f*_1_ and *f*_2_, respectively. The red dashed curve shows the required minimum bandwidth of optoelectronic devices. We assume that the bandwidths of the coding signal and the photodetector (PD) are Δ*f* and *B*, respectively. Therefore, the frequency of the microwave signal generated by beating the optical signals in Fig. 1a should satisfy that *f*_2_ − *f*_1_ ≤ *B* − Δ*f*. This indicates that the highest frequency of generated microwave carrier is *B* − Δ*f*. By contrast, when SSB modulation is used, as shown in Fig. 1b, the microwave frequency after beating satisfies *f*_2_ − *f*_1_ ≤ *B*. As we can see, the highest frequency of the generated microwave is *B*. Therefore, the highest frequency of generated microwave signal is increased by Δ*f* with the same PD. That is to say, the SSB modulation technique can significantly relax the bandwidth requirement of optoelectronic devices, when other devices are the same.

**Fig. 2:**
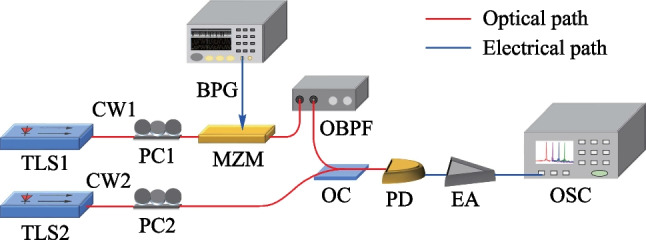
Schematic diagram of the proposed photonic generation of ASK microwave signals with SSB format. TLS: tunable laser source; PC: polarization controller; MZM: Mach–Zehnder modulator; BPG: bit pattern generator; OBPF: optical band pass filter; OC: optical coupler; PD: photodetector; EA: electrical amplifier; OSC: oscilloscope

The schematic diagram of the proposed photonic generation of microwave SSB signals is shown in Fig. 2. A continuous wave (CW1) light generated by a tunable laser source (TLS1) can be expressed as

1 $$E_{1} (t) = E_{1} \cos (\omega_{1} t),$$

where *E*_1_ and *ω*_1_ are the amplitude and angular frequency of CW1, respectively. Then, CW1 is sent into a Mach–Zehnder modulator (MZM), which is biased at the orthogonal transmission point for generating an optical ASK signal. A series of pseudo random binary sequence (PRBS) generated by a bit pattern generator (BPG) is used as the coding signal. Assuming that the period of the PRBS is *T*_0_, the coding signal can be expressed as

2 $$x(t) = \sum\limits_{n} {a_{n} p(t - nT_{0} )} ,$$

where *a*_*n*_ ∈ {− 1, 1}, *p*(*t*) is the unit pulse and the pulse width is *T*_0_. The power spectrum of the PRBS can be expressed as

3 $$X(\omega ) = \frac{1}{{T_{0} }}\left[ {\frac{{\sin (\omega T_{0} /2)}}{\omega /2}} \right]^{2} .$$

According to Eq. (3), the power spectrum of the intensity modulated signal output from MZM can be expressed as

4 $$P_{{{\text{DSB}}}} (\omega ) \propto \frac{1}{{T_{0} }}\left\{ {\frac{{\sin \left[ {(\omega - \omega_{1} )T_{0} /2)} \right]}}{{(\omega - \omega_{1} )/2}}} \right\}^{2} .$$

To obtain an optical SSB signal, an optical band pass filter (OBPF) is used to eliminate the − 1st order sideband of the intensity modulated signal, leaving only the optical carrier and + 1st order sideband. The power spectrum of the optical SSB signal can be expressed as

5 $$P_{{{\text{SSB}}}} (\omega ) \propto \frac{1}{{T_{0} }}\left\{ {\frac{{\sin \left[ {(\omega - \omega_{1} )T_{0} /2)} \right]}}{{(\omega - \omega_{1} )/2}}} \right\}^{2} \cdot {\text{rect}}\left[ {\frac{1}{{\omega_{0} }}\left( {\omega - \omega_{1} - \frac{{\omega_{0} }}{2}} \right)} \right],$$

where rect(*ω*) is a gate function in the frequency domain and *ω*_0_ = 2π/*T*_0_. Then, the optical SSB signal is combined with CW2 emitted from TLS2 by an optical coupler (OC). CW2 can be expressed as

6 $$E_{2} (t) = E_{2} \cos (\omega_{2} t),$$

where *E*_2_ and *ω*_2_ are the amplitude and angular frequency of CW2, respectively. Then, the combined optical signals are sent to a PD to beat with each other. From Eqs. (5) and (6), it can be deduced that the power spectrum of the generated microwave signal can be expressed as

7 $$P (\omega ) \propto \frac{1}{{T_{0} }}\left\{ {\frac{{\sin \left[ {(\omega - \omega_{2}+\omega_{1} )T_{0} /2)} \right]}}{{(\omega - \omega_{2}+ \omega_{1} )/2}}} \right\}^{2} \cdot {\text{rect}}\left[ {\frac{1}{{\omega_{0} }}\left( {\omega - \omega_{2}+\omega_{1} - \frac{{ \omega_{0}}}{2}} \right)} \right].$$

As we can see, the photonically generated ASK microwave signal is in SSB format instead of DSB format, and the microwave carrier angular frequency and coding rate are *ω*_2_ − *ω*_1_ and 1/*T*_0_, respectively. The electrical spectrum occupied by the ASK microwave signal is reduced by half compared with DSB format.

## Experiment

To verify the feasibility of our proposed scheme, an experiment is carried out with the setup shown Fig. 2. Two TLS (NKT Basik E15, ID Photonics CoBrite-DX) are used to generate CW lights. The 3-dB bandwidth of MZM (Fujitsu FTM7938EZ) is 25 GHz. An OBPF (EXFO XTM-50) with an approximate rectangle shape is used to eliminate the −1st order sideband of the optical DSB signal. The high-speed PD (SHF AG Berlin) has a bandwidth of 40 GHz. To boost the microwave power and eliminate the DC component, an electrical amplifier (EA, XJPA1840G3015) with a bandwidth of 18–40 GHz is added after the PD. The PRBS is generated by a BPG (SHF BPG 44E). The signal optical spectrum and eye diagram are measured by an optical spectral analyzer (Yokogawa AQ6370C) and a sampling oscilloscope (Agilent DCA-J 86100C), respectively. The waveform of the microwave SSB signal is measured by a real time oscilloscope (Keysight DSAZ594A) with a sampling rate of 80 GSa/s.

**Fig. 3:**
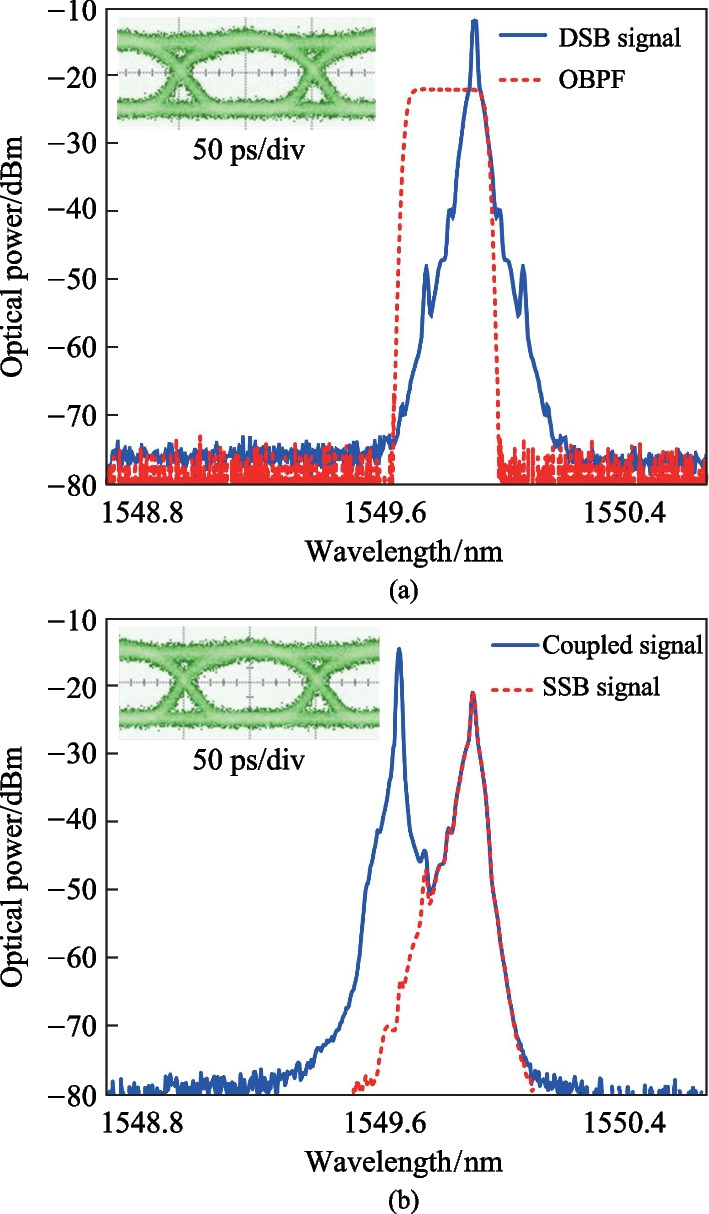
**a** Optical spectrum of the optical DSB signal (blue solid curve) and transmission spectrum of the OBPF (red dashed curve). Inset is the eye diagram of the optical DSB signal. **b** Optical spectrum of the optical SSB signal (red dashed curve) and the optical spectrum after OC (blue solid curve). Inset is the eye diagram of the optical SSB signal

In the experiment, the wavelength and power of CW1 generated by TLS1 are 1549.67 nm and 10 dBm, respectively. After passing through a polarization controller (PC1), CW1 is sent into an MZM. A 2^7^ − 1-bit PRBS signal with a bit rate of 10 Gb/s is generated by the BPG and applied to the MZM, which is biased at the orthogonal transmission point. After MZM, an intensity modulated signal with DSB format is generated, whose optical spectrum is shown by the blue solid curve in Fig. 3a. Additionally, the eye diagram of the optical DSB signal is measured and shown in the inset of Fig. 3a. To obtain an optical SSB signal, an OBPF is used to eliminate the −1st order sideband of the intensity modulated signal, leaving only the optical carrier and the +1st order sideband. The transmission spectrum of the OBPF is shown by the red dashed curve in Fig. 3a. After passing through the OBPF, an optical SSB signal is generated, whose optical spectrum is shown by the red dashed curve in Fig. 3b. As we can see, the − 1st order sideband of the intensity modulated signal has been completely filtered out, which indicates that an optical ASK SSB signal is successfully generated. The measured eye diagram of the optical SSB signal is shown in the inset of Fig. 3b. Then, TLS2 is turned on and the wavelength of CW2 is set to 1549.67 nm. The optical SSB signal and CW2 light are combined by an optical coupler (OC). The measured optical spectrum of the SSB signal and CW2 after OC is shown by the blue solid curve in Fig. 3b. As we can see, the wavelength interval between the two optical carriers is 0.24 nm, corresponding to a frequency interval of 30 GHz. By adjusting the state of polarization of the two CW lights to be aligned with each other, an ASK microwave signal with maximized magnitude is generated after beating at the PD. The generated ASK microwave signal is with SSB modulation format, and the carrier frequency is equal to the frequency interval between the two CW lights.

**Fig. 4:**
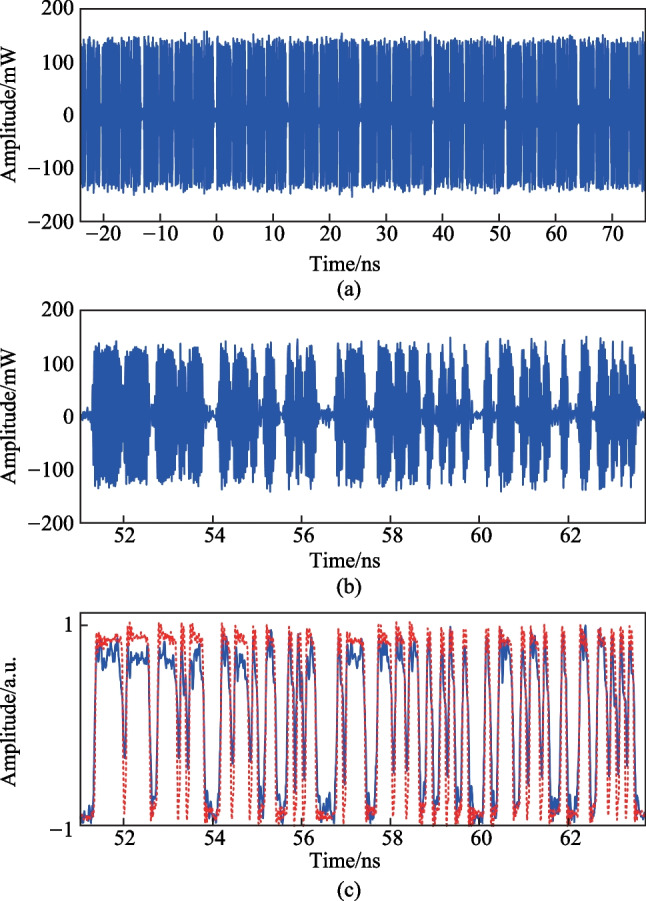
Measured results of the photonically generated microwave signal when the frequency interval between CW1 and CW2 is 30 GHz and the bit rate of the PRBS coding signal is 10 Gb/s. **a** Temporal waveform of the generated microwave SSB signal after the EA. **b** Zoomed-in view of the temporal waveform in one coding period (from 51.05 to 63.75 ns). **c** Recovered coding signal (blue solid curve) and the original PRBS driving signal (red dashed curve)

Figure 4a shows the temporal waveform of the generated microwave SSB signal after the EA in a 100 ns duration time. The carrier frequency of the microwave signal is 30 GHz. The zoomed-in view of the temporal waveform in one coding period (from 51.05 to 63.75 ns) is shown in Fig. 4b. By using the signal envelope extraction method based on Hilbert transform, the waveform of the coding signal (blue solid curve) can be recovered, as shown in Fig. 4c. The original PRBS driving signal (red dashed curve) is also shown in Fig. 4c for comparison. It can be observed that the recovered waveform agrees well with the original PRBS driving signal.

**Fig. 5:**
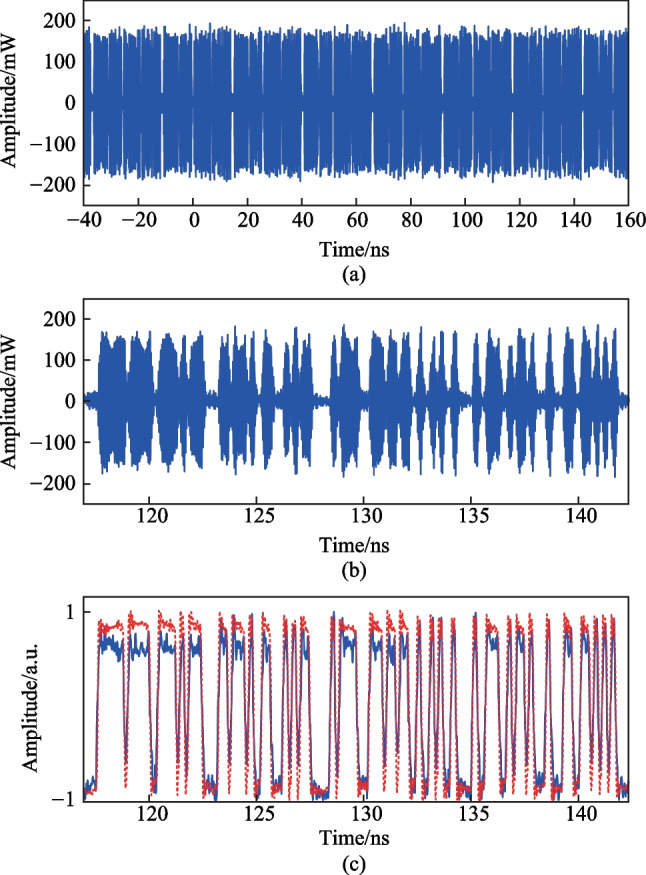
Measured results of the photonically generated microwave signal when the frequency interval between CW1 and CW2 is 20 GHz and the bit rate of the PRBS coding signal is 5 Gb/s. **a** Temporal waveform of the generated microwave SSB signal after the EA. **b** Zoomed-in view of the temporal waveform in one coding period (from 116.95 to 142.25 ns). **c** Recovered coding signal (blue solid curve) and the original PRBS driving signal (red dashed curve)

To demonstrate the flexibility of the generated signal in this scheme, the frequency interval between CW1 and CW2 and the bit rate of the PRBS coding signal are set to 20 GHz and 5 Gb/s, respectively. Figure 5 shows the measured results. Figure 5a shows the waveform of the microwave SSB signal after the EA with a duration time of 200 ns. The zoomed-in view of the temporal waveform in one coding period (from 116.95 to 142.25 ns) is shown in Fig. 5b. The recovered coding signal waveform (blue solid curve) and the original PRBS driving signal (red dashed curve) are shown in Fig. 5c.

In the experiment, it is noticed that two free-running tunable laser sources are used to generate CW lights, whose wavelengths and phases are uncorrelated. Therefore, the carrier frequency drift of the generated microwave signal originates from the wavelength drifts of two lasers. Additionally, the relative phase jitter between two lasers can also lead to unstable phase of the generated microwave signal. These problems can degrade microwave waveform and increase bit error rate in microwave communications. To solve these problems, optical frequency comb, optical phase-locked loop, or optical injection locking can be used to obtain two optical signals with correlated wavelengths [5].

To further increase the spectrum efficiency, the proposed scheme can be combined with advanced modulation formats, such as quadrature amplitude modulation (QAM). Assuming that the baseband signal has a period of *T*_0_, the optical QAM signal can be expressed as

8 $$E_{{{\text{QAM}}}} (t) = E_{1} \sum\limits_{n} {A_{n} p(t - nT_{0} ) \cdot } \cos (\omega_{1} t - \varphi_{n} ),$$

where *A*_*n*_ is the amplitude of the baseband signal. The I branch signal and Q branch signal can be obtained by mathematical transformation.

9 $$\begin{aligned} E_{{{\text{QAM}}}} (t) &= E_{1} \left\{ {\sum\limits_{n} {\left[ {A_{n} p(t - nT_{0} )\cos \varphi_{n} } \right]} \cos (\omega_{1} t)} \right. \\ &\,\,\,\,\,+ \left. {\sum\limits_{n} {\left[ {A_{n} p(t - nT_{0} )\sin \varphi_{n} } \right]} \sin (\omega_{1} t)} \right\} \\ &= E_{1} \left[ {\sum\limits_{n} {i_{n} p(t - nT_{0} )} \cos (\omega_{1} t)} \right. \\ &\,\,\,\,\,+ \left. {\sum\limits_{n} {q_{n} p(t - nT_{0} )} \sin (\omega_{1} t)} \right] \\ &= E_{1} \left[ {I(t)\cos (\omega_{1} t) + Q_{1} \sin (\omega_{1} t)} \right], \\ \end{aligned}$$

where *i*_*n*_ = *A*_*n*_cos*φ*_*n*_ and *q*_*n*_ = *A*_*n*_sin*φ*_*n*_ give the constellation points in the QAM signal constellation diagram. As we can see, the QAM signal combines two amplitude modulation signals, which have the same carrier frequency and 90° phase difference. Therefore, in the frequency domain, the optical QAM signal is a DSB signal, whose sidebands are symmetric about *ω*_1_. After eliminating the − 1st order sideband of the QAM signal, an optical QAM signal with SSB format can be obtained. By beating the optical SSB signal with CW2 light, a QAM microwave signal with SSB format is generated. Therefore, our proposed approach can also be used to generate the QAM microwave signal with SSB format.

## Conclusion

We have proposed and experimentally demonstrated the photonic generation of microwave SSB signals by beating a CW optical signal with an SSB optical signal. In the experiment, an ASK microwave signal with SSB format is generated with a carrier frequency of 30 GHz and a coding bit rate of 10 Gb/s. To demonstrate the flexibility of the generated signal in this scheme, the carrier frequency and the coding rate of the ASK microwave SSB signal are adjusted to 20 GHz and 5 Gb/s, respectively. The microwave SSB signal can effectively avoid the problem of power attenuation that can be introduced by chromatic dispersion in optical fiber transmission. It can also relax the bandwidth requirement of optoelectronic devices in microwave communication systems.

## Data Availability

The data that support the findings of this study are available from the corresponding author, upon reasonable request.
